# Analgesic efficacy of an opioid-free postoperative pain management strategy versus a conventional opioid-based strategy following laparoscopic radical gastrectomy: an open-label, randomized, controlled, non-inferiority trial

**DOI:** 10.1186/s12957-023-03298-x

**Published:** 2024-02-15

**Authors:** Zhimin Lin, Zhongbiao Chen, Yongliang Li

**Affiliations:** 1 Fuzong Clinical Medical College, Fujian Medical University, Fuzhou, 350001 People’s Republic of China; 2 The 900th, Hospital of Joint Logistic Support Force, PLA, Fuzhou, 350001 People’s Republic of China; 3 Affiliated Hospital of Putian University, 999th Dongzhen East Road, Licheng District, Putian, 351100 People’s Republic of China

**Keywords:** Regional anesthesia, Ultrasound, Continuous subcostal transversus abdominis plane block, Laparoscopic radical gastrectomy, Opioid-free

## Abstract

**Objective:**

In patients undergoing laparoscopic radical gastrectomy, the use of subcostal transversus abdominis plane block (STAPB) for completely opioid-free postoperative pain management lacks convincing clinical evidence.

**Methods:**

This study included 112 patients who underwent laparoscopic radical gastrectomy at the 900TH Hospital of the Joint Logistics Support Force from October 2020 to March 2022. Patients were randomly divided into (1:1) continuous opioid-free STAPB (C-STAPB) group and conventional group. In the C-STAPB group, 0.2% ropivacaine (bilateral, 20 ml per side) was injected intermittently every 12 h through a catheter placed on the transverse abdominis plane for postoperative pain management. The conventional group was treated with a conventional intravenous opioid pump (2.5 μg/kg sufentanil and 10 mg tropisetron, diluted to 100 ml with 0.9% NS). The primary outcomes were the accumulative area under the curve of the numeric rating scale (NRS) score at 24 and 48 h postoperatively at rest and during movement. The secondary outcomes were postoperative recovery outcomes, postoperative daily food intake, and postoperative complications.

**Results:**

After exclusion (*n* = 16), a total of 96 patients (C-STAPB group, *n* = 46; conventional group, *n* = 49) were included. We found there were no significant differences in the cumulative AUC of NRS score PACU-24 h and PACU-48 h between the C-STAPB group and conventional group at rest [(mean difference, 1.38; 95% CI, − 2.21 to 4.98, *P* = 0.447), (mean difference, 1.22; 95% CI, − 6.20 to 8.65, *P* = 0.744)] and at movement [(mean difference, 2.90; 95% CI, − 3.65 to 9.46; *P* = 0.382), (mean difference, 4.32; 95% CI, − 4.46 to 13.1; *P* = 0.331)]. The 95% CI upper bound of the difference between rest and movement in the C-STAPB group was less than the inferior margin value (9.5 and 14 points), indicating the non-inferiority of the analgesic effect of C-STPAB. The C-STAPB group had faster postoperative recovery profiles including earlier bowel movement, defecation, more volume of food intake postoperative, and lower postoperative nausea and vomiting compared to conventional groups (*P* < 0.001).

**Conclusions:**

After laparoscopic radical gastrectomy, the analgesic effect of C-STAPBP is not inferior to the traditional opioid-based pain management model.

**Trial registration:**

ChiCTR2100051784.

## Introduction

Gastric cancer is an important global disease, mainly distributed in East Asia, Eastern Europe, and South America [1]. It is estimated that there are more than 1 million new cases of gastric cancer each year, which is the fifth largest malignant tumor in the world [1]. At present, laparoscopic surgery has become the mainstream of gastric cancer surgery. Although laparoscopic surgery has significantly reduced the patient’s physical trauma, there are still 5–10-cm surgical incisions, and postoperative pain is still serious [2]. Opioids are still the cornerstone of anesthesia and postoperative pain management in laparoscopic radical gastrectomy. However, because opioid receptors are widely present in various organs of the human body, they can also cause opioid-related adverse events (ORAE), such as nausea, vomiting, dizziness, intestinal paralysis, and respiratory depression while exerting analgesic effects [3].

Related studies have shown that the application of a large number of opioids during the perioperative period would significantly prolong the length of hospital stay [4]. According to statistics, the average incidence of ORAE was as high as about 20%, and the incidence of ORAE after gastrointestinal surgery was higher [4]. Therefore, it is important to minimize the use of opioids after gastrointestinal surgery. The postoperative analgesia model opioid-free is ideal, but it still needs to be studied.

Transversus abdominis plane block (TAPB) is the most common regional anesthesia used to manage postoperative pain after abdominal surgery [5–7]. However, this technique provides a limited duration of postoperative analgesia (last 8–12 h) [8]. Compared with single injection STAPB, continuous transversus abdominis plane block (C-STAPB) can reduce postoperative pain and opioid dosage [8]. In various abdominal surgeries, C-STAPB can be used as a feasible alternative to subdural catheter block and is a safe and effective analgesic method [9]. At present, there is no relevant study to compare the analgesic effect of patient-controlled intravenous analgesia pump (PCIA) and C-STAPB after laparoscopic radical gastrectomy. Most importantly, as far as we know, whether C-STAPB can achieve complete opioid-free postoperative pain management in patients undergoing laparoscopic radical gastrectomy remains to be studied.

Therefore, we designed a C-STAPB procedure to achieve complete opioid-free postoperative pain management. In this randomized controlled trial, bilateral STABP was performed simultaneously with continuous intermittent injection of a long-half-life local anesthetic (ropivacaine). The purpose of this study was to evaluate the non-inferiority of opioid-free C-STAPB in pain management after laparoscopic radical gastrectomy compared with traditional opioid-based regimens.

## Materials and methods

### Trial design

This open-label, randomized, controlled, non-inferiority trial was approved by the 900TH Hospital of the Joint Logistics Support Force Institutional Review Board (2,014,046) and conducted in accordance with the Helsinki Declaration. This study follows the comprehensive standard guidelines for test reports. Written informed consent of all patients was obtained. This study was registered in ChiCTR.org.cn (ChiCTR2100051784).

### Participants

This study was conducted from October 2020 to March 2022 at the 900TH Hospital of the Joint Logistics Support Force, one of the largest cancer centers in southeastern China. The sample size was estimated using PASS software, because there were not enough relevant references in the literature at the time. As advised, the noninferiority was established at 1/5 of the conventional group mean [10, 11]. The cumulative AUCs of PACU-24 h and PACU-48 h at the movement of the ten attempts of conventional procedures were 48.20 ± 16.20 and 86.40 ± 42.26. Based on this, we determined that the non-inferiority values of the average cumulative AUC of PACU-24 h and PACU-48 h at movement were 9.5 and 14 points. Based on that, a total of 100 patients were calculated (1:1 ratio) to be needed (50 in each group) for the primary analysis at rest, with a significance level of one side *α* = 0.025, a power of 1 − *β* = 0.85, and 25% contingency factor. Similarly, a total of 60 patients were calculated (1:1 ratio) to be needed (30 in each group) for the primary analysis at movement.

Inclusion criteria are as follows: (1) the diagnosis of the gastric malignant tumor was confirmed by CT, MRI, gastroscopy, and pathological diagnosis; (2) preoperative evaluation of lymph node metastasis without organ and distant metastasis and in line with laparoscopic surgery; (3) American Society of Anesthesiologists (ASA) grade I/III; (4) age 18–75 years old.

Exclusion criteria are as follows: (1) unable to cooperate with the experiment due to language communication disorders or severe cognitive impairment; (2) allergic to drug addicts or experimental-related drugs; (3) other surgical history.

### Randomization and masking

Patients were randomly divided into the C-STAPB group or conventional group at a ratio of 1:1. Each patient was randomly grouped by a third-party professional medical staff through a computer-generated random number. The odd random number was the conventional group, and the even random number was the C-STAPB group. For ethical reasons, patients and doctors who took care of the patients since intraoperatively and postoperatively did not blind the patient allocation group.

### Interventions

**Fig. 1:**
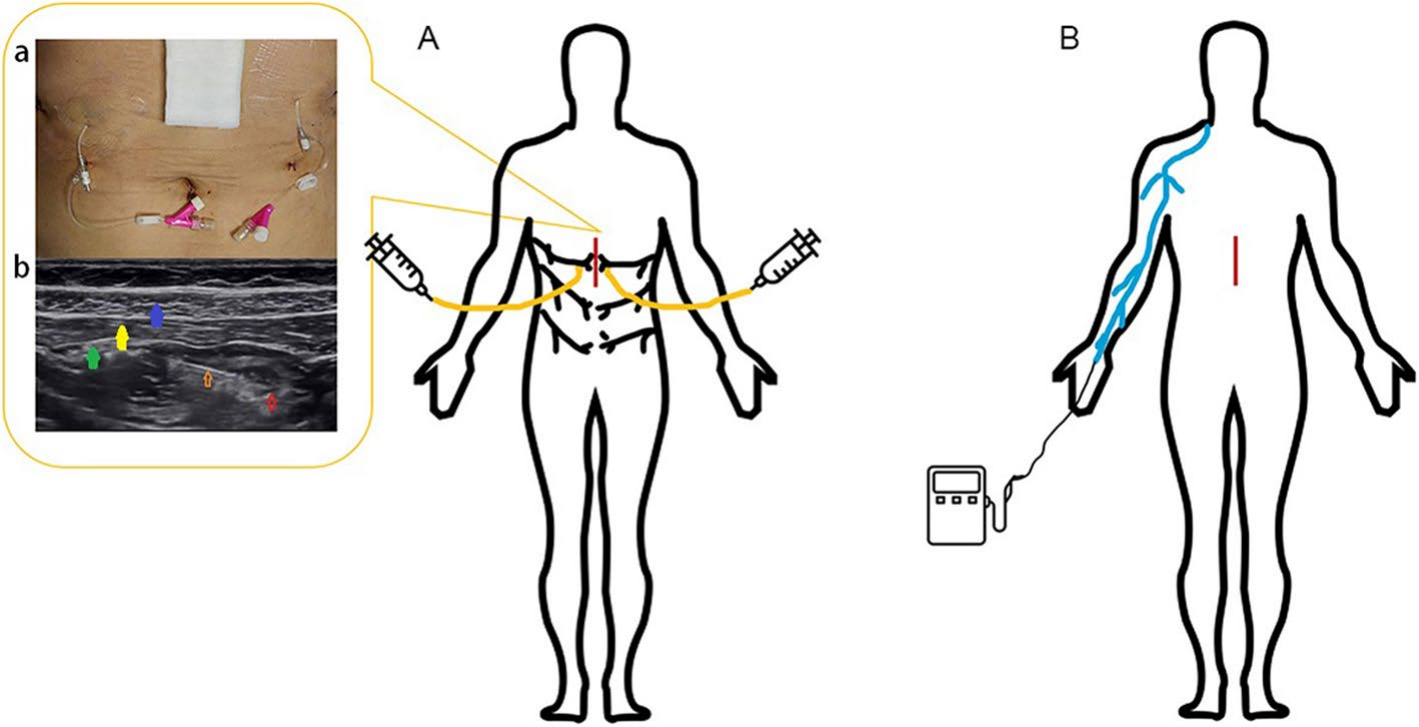
Trial intervention diagram. **A** The intervention of the C-STAPB group. **B** The intervention of the conventional group. (a) Bilateral transverse abdominal muscle plane catheterization. (b) Transversus abdominis plane block under ultrasound (red arrow for local anesthetic, orange arrow for the needle, blue arrow for the external oblique, yellow arrow for the internal oblique, green arrow for the transversus abdominis muscle). Abbreviations: C-STAPB, continuous subcostal transversus abdominis plane block

In the C-STAPB group, after the abdominal incision was closed, the transverse abdominal plane block catheter was established on the lateral side of the bilateral meniscus of the surgical incision. Using ultrasonic positioning, the trocar was inserted from the tail end to the head end into the gap between the internal oblique muscle and the transverse abdominal muscle. 5 ml 0.9% NS was injected to expand the space, and then the trocar was pushed about 0.5–1.0 cm to ensure stability and reliability. Then, the hard needle was withdrawn, the catheter was fixed locally with a transparent paste, and the length of catheter exposure was recorded. After confirming the appropriate position of the catheter, the first dose was injected (40 mL 0.2% ropivacaine, 20 mL on each side). Ultrasound was used to observe the liquid diffusion in the injection space. Repeat the same procedure on the opposite side. In the C-STAPB group, the drug was administered once every 12 h after surgery through a fixed catheter until 48 h after surgery in the post-anesthesia care unit (PACU) (20 ml per side, 5 postoperative injections). In the conventional group, patients received a conventional opioid PCIA pump (2.5 μg/kg sufentanil and 10 mg tropisetron, diluted to 100 mL with 0.9% NS). Considering the strict bias control, all intravenous pumps were locked to administer only the basic dose (2 mL/h), of which patient-controlled additional use was disabled (Fig. 1).

All patients underwent general anesthesia by the same experienced anesthesiologist and the same anesthesia regimen was used. All surgeries are performed by experienced gastrointestinal surgeons. Postoperative pain numeric rating scale (NRS, score: 0–10, the higher the score, the more severe the pain) was used to assess the pain in the first 4 days after surgery. The decision to provide rescue analgesia was made by the surgeon and was only considered when the patient requests and the NRS ≥ 4. Flurbiprofen (50 mg) was the only non-steroidal anti-inflammatory drug (NSAID) used to rescue analgesia. The C-STAPB catheter and PCIA pump were removed 48 h after the operation.

### Outcomes

#### The primary outcomes

The primary outcomes included the cumulative area under the curve (AUC) of the pain NRS score at 24 and 48 h after surgery both at rest and during movement. Pain score at rest and movement at 24 and 48 h was assessed using the numeric rating scale from 0 to 10, According to the NRS score and postoperative time, the overall AUC of pain score at rest and during movement at 48 h after surgery was compared. The time points were set at PACU (0 h), 24 h, and 48 h after leaving PACU. The calculation formula of AUC is as follows: AUC = ∑ (NRS_i_ + NRS_j_) * (*t*_*j*_ − *t*_*i*_)/2, when NRS_i_ and NRS_j_ were the NRS scores corresponding to two adjacent observation time points *t*_*j*_ and *t*_*i*_ (*j* > *i*), respectively.

#### The secondary outcomes

The secondary outcomes included the proportion of patients requiring rescue analgesia, postoperative complications, bowel recovery functions, time to first defecation, bowel movement and daily food intake, recovery by time to start ambulation, length of hospital stay, STAPB-related events, and surgical complications. All patients took 40 ml meglumine diatrizoate orally on the second day after the operation, and the time of the radiography agent (meglumine diatrizoate) reaching the ileocecal junction was recorded.

### Statistical analysis

All statistical analyses were performed using SPSS 11.0 software. Continuous variables are expressed as mean (SD) or median [interquartile range (IQR)] and compared using Student’s *t* test or the Mann–Whitney *U* test, as appropriate. Categorical variables are expressed as absolute frequencies (percentages) and compared using Pearson’s *χ*^2^ test or Fisher’s exact test, as appropriate. Student’s *t* test was used to analyze the NRS scores of the two groups at each time point. Repeated measurement analysis of variance was used for statistical analysis of measurement data that were repeated for the same individual and conformed to the normal distribution. For the primary outcomes, the difference with the 95% confidence interval [CI] is calculated to estimate the non-inferiority. And the non-inferiority values of the average cumulative AUC of PACU-24 h and PACU-48 h were 9.5 and 14 points.* P* < 0.05 was considered statistically significant.

## Results

### Basic information of patients

**Fig. 2:**
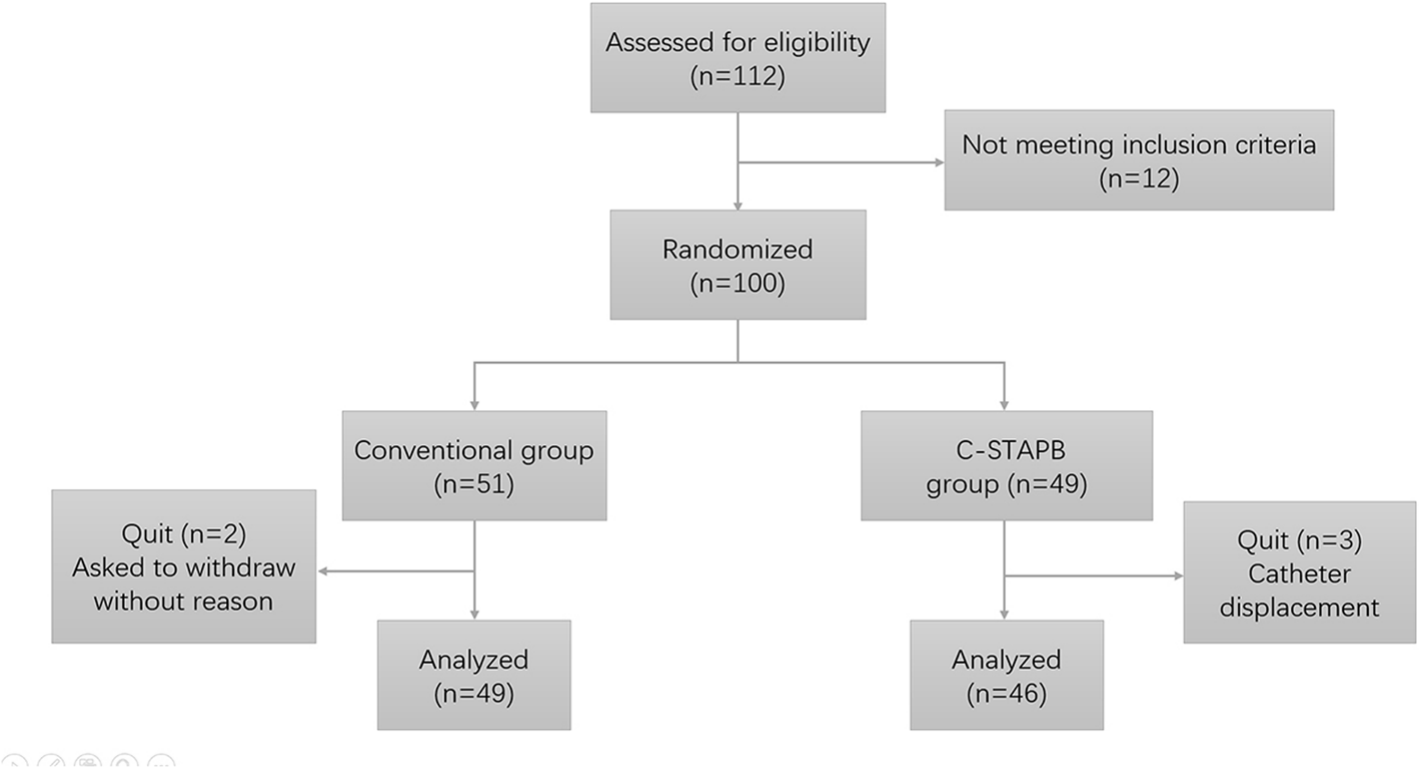
CONSORT flow diagram

Of the 112 patients screened during the study period, 100 were finally enrolled and randomly assigned to each group (C-STAPB group 49, conventional group 51). In the conventional group, two patients were asked to withdraw from the experiment without reason, and in the C-STAPB group, three patients withdrew from the experiment due to catheter displacement (Fig. 2). The results showed that there was no significant difference in gender, age, body mass index (BMI), preoperative nutritional score, and pathological stage between the two groups (*P* > 0.05). Although the proportion of laparoscopic radical total gastrectomy in the C-STAPB group was higher than that in the conventional group (58.70% vs 42.86%, *P* > 0.05), the difference between the two groups was not statistically significant (Table 1).

**Table 1:** General information of the two groups

Characteristic	C-TAPB group (*n* = 46)	Conventional group (*n* = 49)	*P* value
Sex, No. (%)			0.38
Male	27 (58.70)	33 (67.35)	
Female	19 (41.30)	16 (32.65)	
Age, mean (SD), y	59.20 ± 11.92	60.33 ± 11.22	0.51
Height, mean (SD), cm	164.07 ± 7.88	163.88 ± 7.51	0.43
Weight, mean (SD), kg	61.59 ± 11.07	59.85 ± 10.32	0.57
BMI, mean (SD), kg/m2	22.76 ± 2.99	22.20 ± 3.02	0.75
Nutrition score, No. (%)			0.26
< 3	36 (78.26)	38 (77.55)	
≥ 3	10 (21.74)	11 (22.45)	
AJCC stage, No. (%)			0.90
I	15 (32.61)	14 (28.57)	
II	11 (23.91))	13 (26.53)	
III	20 (43.48)	22 (44.90)	
Surgical procedure, No. (%)			0.12
Radical distal subtotal gastrectomy	19 (41.30)	28 (57.14)	
Radical total gastrectomy	27 (58.70)	21 (42.86)	
Length of abdominal incision, mean (SD), cm	8.63 ± 1.02	8.55 ± 0.91	0.42
Time of surgery, (SD), min	236.80 ± 24.63	232.88 ± 19.26	0.57
Intraoperative blood loss, (SD), mL	69.13 ± 30.25	72.65 ± 27.60	0.15
Intraoperative infusion volume, (SD), mL	1600.00 ± 508.4	1469.34 ± 406.31	0.19

*BMI* body mass index, *AJCC* American Joint Committee on Cancer

### Cumulative AUC of postoperative NRS

We found no significant differences in the cumulative AUC PACU-24 h and PACU-48 h between the C-STAPB group and conventional group at rest [(mean difference, 1.38; 95% CI, − 2.21 to 4.98, *P* = 0.447), (mean difference, 1.22; 95% CI, − 6.20 to 8.65, *P* = 0.744)] and at movement [(mean difference, 2.90; 95% CI, − 3.65 to 9.46; *P* = 0.382), (mean difference, 4.32; 95% CI, − 4.46 to 13.1; *P* = 0.331)] (Table 2).

**Table 2:** The primary outcomes, mean (SD), score

Characteristic	C-STAPB group *(n* = 46)	Conventional group (*n* = 49)	Difference (95% CI)	*P* value
**AUC at rest**				
PACU-24 h	26.61 ± 9.77	25.22 ± 7.84	1.38 (− 2.21, 4.98)	0.447
PACU-48 h	36.00 ± 20.08	34.78 ± 16.29	1.22 (− 6.20, 8.65)	0.744
**AUC at movement**				
PACU-24 h	51.39 ± 16.13	48.49 ± 16.05	2.90 (− 3.65, 9.46)	0.382
PACU-48 h	86.61 ± 21.75	82.29 ± 21.35	4.32 (− 4.46, 13.10)	0.331

*C-STAPB* continuous transversus abdominis plane block, *CI* confidence interval, *AUC* area under the curve, *PACU* post-anesthesia care unit

At rest and movement, there was no significant difference in NRS between the C-STAPB group and the conventional group at 0 h, 24 h, and 48 h after surgery (*P* = 0.80 > 0.05) (*P* = 0.25 > 0.05). Similarly, there was no significant difference in the rate of patients who required rescue analgesia between the two groups (*P* > 0.05) at postoperative 24 and 48 h (Table 3).

**Table 3:** NRS and number of patients who required rescue analgesia

Characteristic	C-STAPB group (*n* = 46)	Conventional group (*n* = 49)	*P* value
**NRS at rest**			0.80^*^
NRS-PACU	1.33 ± 0.60	1.27 ± 0.45	0.12
NRS-24 h	0.89 ± 0.38	0.84 ± 0.37	0.47
NRS-48 h	0.17 ± 0.38	0.18 ± 0.39	0.81
**NRS at movement**			0.25^*^
NRS-PACU	2.44 ± 0.66	2.33 ± 0.75	0.27
NRS-24 h	1.85 ± 0.82	1.71 ± 0.74	0.45
NRS-48 h	1.17 ± 0.44	1.10 ± 0.31	0.06
**Number of patients who required rescue analgesia* n* (%)**			
PACU-24 h	42 (91.3)	41 (83.7)	0.42
24–48 h	32 (69.6)	33 (67.3)	0.82
48–72 h	27 (58.7)	24 (49.0)	0.34

^*^P-value between groups using repeated measures analysis of variance

*NRS* numeric rating scale, *C-STAPB* continuous transversus abdominis plane block, *PACU* post-anesthesia care unit

### The secondary outcomes

Postoperative recovery outcomes include time of defecation, bowel movement, time of off-bed, postoperative length of stay, and postoperative daily food intake. The time of defecation (29.56 ± 5.33 vs 50.12 ± 3.90, *P* = 0.03) and the bowel movement (time of radiography agent to ileocecal junction) (1.76 ± 0.26 vs 2.10 ± 0.39, *P* = 0.01) in the C-STABP group were significantly earlier than those in the conventional group. In addition, there was a statistically significant difference in postoperative daily food intake between the C-STABP group and the conventional group (*P* < 0.001) (Table 4).

**Table 4:** Secondary outcomes

Secondary outcomes	C-STAPB group (*n* = 46)	Conventional group (*n* = 49)	*P* value
**Postoperative recovery outcomes**			
Time of defecation, mean (SD), h	29.56 ± 5.33	50.12 ± 3.90	0.03
Bowel movement, mean (SD), h	1.76 ± 0.26	2.10 ± 0.39	0.01
Off-bed, mean (SD), day	2.17 ± 0.64	2.00 ± 0.58	0.17
PLOS, mean (SD), day	8.96 ± 1.63	9.43 ± 2.03	0.44
**Postoperative daily food intake**			< 0.001
Day 1, mean (SD), ml	64.24 ± 60.82	20.10 ± 19.86	< 0.001
Day 2, mean (SD), ml	200.98 ± 121.75	65.31 ± 25.83	< 0.001
Day 3, mean (SD), ml	457.17 ± 218.90	151.53 ± 58.09	< 0.001
Day 4, mean (SD), ml	713.63 ± 234.37	280.72 ± 106.26	< 0.001
**Postoperative complication**			
Flush	0 (0)	0 (0)	1.00
Nausea or vomit	3 (6.52)	11 (22.45)	0.03
Dizziness	6 (13.04)	9 (18.37)	0.45
Bradycardia	0 (0)	0 (0)	1.00
Pneumonia	2 (4.35)	1 (2.04)	0.52
Delirium	0 (0)	0 (0)	1.00
Spasticity	0 (0)	0 (0)	1.00
Retention of urine	1 (2.17)	1 (2.04)	0.96
Other surgical complications	2 (4.35)	3 (6.12)	0.70

*C-STAPB* continuous transversus abdominis plane block, *PLOS* postoperative length of stay

The incidence of postoperative nausea and vomiting in the C-STABP and conventional groups was significantly different (*p* = 0.03), but there was no significant difference in other complications between the two groups (Table 4).

## Discussion

Additional costs associated with various ORAE and complications increase the medical burden, especially for gastrointestinal surgery [12]. Therefore, such patients urgently need an ideal method to reduce postoperative opioid requirement for pain management. To the best of our knowledge, this is the first study to report that C-STAPB is not inferior to traditional opioids in pain management after laparoscopic radical gastrectomy [13–15]. We found that it is feasible to achieve the goal of opioid-free postoperative pain management. In addition, the study design of this study is rigorous and innovative.

The pain after abdominal surgery mainly comes from abdominal wall pain, which can last for 2–3 days [16]. Therefore, good abdominal wall analgesia is particularly important. According to studies, patients who utilized TAPB in conjunction with general analgesia could reduce opioid consumption by 36% [17]. TAPB combined with intravenous opioid analgesia was not inferior to thoracic epidural analgesia in the pain effects after abdominal surgery, but the incidence of postoperative nausea, vomiting, intestinal obstruction, and paresthesia was lower than that of thoracic epidural analgesia [18]. The duration of a single-injection TAPB generally does not exceed 12 h [19]. But the C-STAPB procedure used a long-term bilateral catheter and repeated intermittent injections of ropivacaine, which greatly prolonged the analgesic effect [20, 21]. In this study, there was no significant difference in the pain score at rest and movement, Moreover, the number of patients that require rescue analgesia between the C-STABP group and the conventional group indicates that there was no significant difference in the analgesic effect between the two groups. C-STAPB can achieve effective postoperative pain management with opioid-free.

Here, we demonstrate that C-STAPB is beneficial to the postoperative recovery of patients compared to the conventional group and reduces postoperative opioid-related adverse reactions. Our results showed that the C-STAPB group had earlier bowel recovered function in aspects of bowel movement, first defecation, and food intake compared to the conventional group. The fundamental reason for this result is that the patients in the C-STAPB group did not use opioids after surgery [4, 17]. In contrast to Stoner et al.’s findings, neither the incidence of dizziness nor the time off-bed differed significantly between the C-STAPB group and the conventional group [17]. Additionally, the C-STAPB group had a shorter length of stay in the hospital after surgery than the conventional group; the difference was not statistically significant. This occurrence may be caused by the fact that these indicators are strongly influenced by the patients’ and researchers’ own subjective personal variables.

The most common complications associated with TSAPB involve injection needles and regional anesthetics procedure. According to reports, situating the lower transverse abdominis plane block using body surface anatomical landmarks can accidentally penetrate the abdominal cavity and might injure the visceral organs [22]. With the development and application of ultrasound technology, the incidence of adverse events such as local hematoma and abdominal organ injury has been greatly reduced. Regional anesthetic-related complications of STAPB include seizures from local anesthetic systemic toxicity (LAST), ventricular arrhythmias, and transient nerve paralysis [23, 24]. When the block is required a high volume of local anesthetic drugs, a low concentration with an increased volume of drug should be considered for regional anesthesia. Therefore, ropivacaine, which has little effect on the central nervous system and cardiovascular and cerebrovascular system, is selected as a regional anesthetic, and it is less likely to cause adverse reactions such as hypertension, cardiac arrest, respiratory depression, and convulsions [25].

Since the analgesic effect of STAPB is based on the degree of spreading of the local anesthetic drug, the volume of the local anesthetic drug is the primary factor affecting the process of the block [25]. Studies have shown that there wasno advantage of increasing the local anesthetic drug greater than 20 ml for STAPB [25]. At present, there are two main methods of administration for C-STAPB: one is low-dose continuous infusion, and the other is repetitive intermittent administration [26, 27]. Compared with continuous infusion, an intermittent bolus of ropivacaine was found to not only lessen the overall local anesthetic requirement per day, but also has a wider sensory dermatome blockade and longer duration of analgesia.

The current research has several limitations. First of all, C-STAPB is troublesome to implement and requires good patient compliance. We will further simplify the procedure. Secondly, only patients undergoing laparoscopic radical gastrectomy for gastric cancer were included in this study. Whether these findings can be extended to other abdominal surgery remains to be evaluated. Thirdly, in the course of the experiment, three patients did withdraw from the experiment due to catheter displacement (6%), and the way of catheterization should be improved in further experiments. Fourthly, the study did not use patients control analgesia to measure the exact rescue analgesic requirement. The prn dose of rescue analgesic requirement by the nurses might be some barrier between patients and nurses or some factors that cause patients to not receive rescue analgesic drug. Finally, this is a single-center randomized controlled trial, and future studies with multiple centers and larger sample sizes are needed to verify the results of the study.

## Conclusion

In conclusion, our results suggest that C-STAPB is not inferior to traditional opioid-based postoperative pain management for patients undergoing laparoscopic radical gastrectomy, suggesting that it is feasible to achieve complete opioid-free postoperative pain management. C-STAPB is a safe and feasible method with better sequelae, which accelerates the postoperative recovery of patients undergoing laparoscopic radical gastrectomy.

## Data Availability

Data are available with publication from the corresponding author upon reasonable request.
